# Proceedings of the American Dental Association

**Published:** 1860-10

**Authors:** 


### Proceedings of Societies.

### PROCEEDINGS OF THE AMERICAN DENTAL ASSOCIATION.

The delegates of the various local dental Associations and Colleges met according to adjournment, in the Smithsonian Institute, in Washington City, D. C., at 12 o'clock, M., July 31, 1860.

The President, Dr. W. W. Allport, on taking the chair, made the following remarks:

Gentlemen:—Something over a year ago it was proposed by several eminent practitioners of Dental Surgery, that the interests of our profession would be promoted by the formation of a National Dental Association upon a representative basis. To ascertain the opinion of some of the leading Dentists in this country as to the expediency of forming such an association, a memorial was sent to several of our principal cities, for the signatures of those who approved the formation of the proposed association.

The memorial referred to reads as follows:

The undersigned practitioners of Dentistry, believing that a National Association of Dentists, composed of delegates from State, county and local Societies and dental Colleges, would be calculated to promote the best interests of the profession, respectfully suggest to the Dental Societies and Colleges throughout the country, the propriety of electing delegates to meet in Convention at the Falls of Niagara on the first Wednesday of August, 1859, for the purpose of forming, if the assembled delegates shall deem it expedient, a National Association upon a representative basis.

In a few weeks there were names enough attached to this memorial to warrant its publication in the journals of the profession. In compliance with it, two of the Dental Colleges, and the Societies then existing, elected delegates, to meet in Convention, for the object, and at the time and place proposed. At the appointed time, the delegates met, but after mature deliberation, it was concluded that it would be better to postpone the final action in regard to the matter for one year.

A Constitution, however, was drawn up, and has been published in our Dental Journals, which is now to be submitted to you for your consideration.

By some, it may be supposed that it is the wish of the committee who submitted this Constitution, that it be adopted at the present time, without any *material* change, *nolens volens.* I have reason to believe that this is not the case, but that it is the desire of the committee that every section of it be carefully and thoroughly discussed, without fear or favor, and that it be adopted, wholly or in part, or rejected entirely and a new one substituted in its stead, as it may seem to you the best calculated to promote the interests of the profession.

Trusting that the Chair may receive your kind and cordial support, without which our labors would result in discord and unprofitableness, and hoping that a spirit of trust and harmony may pervade all our deliberations, and guide us to fortunate results therein, we will now proceed to the business for which we are assembled.

The Minutes of the preliminary meeting were read, amended and approved.

On motion of Dr. Barker,

*Resolved,* That a committee of five be appointed to examine and report on credentials.

The Chair appointed upon that committee, Drs. Geo. T. Barker, F. Y. Clark, C. P. Fitch, A. Blake, P. G. C. Hunt.

On motion, adjourned to meet at 4 o'clock, P. M.

4 O'CLOCK, P. M.

The Association met according to adjournment.

The Committee on Credentials made the following report:

GENTLEMEN:—The Committee on Credentials respectfully report, that upon examination, the following gentlemen were duly elected delegates to this Convention:

*Washington City Dental Association.*—Drs. J. B. Gibbs and H. W. Wadsworth.

*Georgia Dental Association, Savannah.*—Dr. F. Y. Clark.

*Cincinnati Dental Association.*—Drs. J. Taft and H. R. Smith.

*Mad River Valley Dental Society.*—Dr. Wm. A. Pease.

*Pennsylvania Association of Dental Surgeons.*—Drs. S. Dillingham, J. H. McQuillen, J. Foster Flagg, C. P. Fitch, J. W. Van Osten, J. McFarland, B. M. Gildea and Geo. T. Barker.

*Pennsylvania College of Dental Surgery.*—Dr. J. L. Suesserott.

*Western Dental Society.*—Drs. W. W. Allport and A. Blake.

*Indiana State Dental Association.*—Drs. P. G. C. Hunt and S. B. Smith.

*Kentucky State Dental Association.*—Drs. W. M. Rogers and A. S. Talbert.

*Mississippi Valley Dental Association.*—Dr. W. H. Atkinson.

*Michigan Dental Association.*—Dr. Wm. Cahoon.

GEO. T. BARKER,
C. P. FITCH,
AARON BLAKE,
P. G. C. HUNT,
F. Y. CLARK,
*Committee on Credentials.*

The Committee appointed to prepare a draft of a Constitution was called upon, and made a report in accordance with their appointment.

On motion,

*Resolved*, That the draft of a Constitution presented by the committee be accepted, and the committee discharged.

*Resolved*, That the Constitution be taken up article by article, seriatim, considered, and disposed of.

After being duly considered and amended, it was adopted, and is as follows :

### CONSTITUTION.

### ARTICLE I.—NAME.

This organization shall be known by the name of the *American Dental Association.*

### ARTICLE II.—OBJECTS.

The objects of this Association shall be, to cultivate the science and art of dentistry, and all its collateral branches, to elevate and sustain the professional character of dentists, to promote among them mutual improvement, social intercourse, and good feeling, and to collectively represent and have cognizance of the common interests of the dental profession in every part of the United States.

### ARTICLE III.—MEMBERS.

SECTION I.—The members of this Association shall be exclusively practitioners of dentistry, holding their appointment to membership either as delegates from local institutions, or as permanent members.

SEC. II.—The delegates shall receive their appointment from permanently organized dental societies and dental colleges in the Union, each delegate holding his appointment for one year.

SEC. III.—Each local society shall be entitled to send to the Association, one delegate for every five of its active members, and the faculty of each college to send one of its members as a representative.

SEC. IV.—The permanent members shall consist of all those who have served in the capacity of delegates. Each delegate and permanent member shall be entitled to debate and vote on all questions, and be eligible to any office in the gift of the Association.

SEC. V.—To defray the expense of printing the Transactions, and to meet other incidental expenses, the sum of two dollars shall be assessed annually upon all the members present at meetings of the Association. No member who is not present at a meeting of the Association shall be required to pay the annual assessment.

SEC. VI.—Each member elect, prior to the permanent organization of the annual meeting, or before voting on any question after the meeting has been organized, must sign these regulations, inscribing his name in full, specifying in what capacity he attends, and if a delegate, the title of the institution from which he has received his appointment.

SEC. VII.—No one shall be permitted to address the association before giving his name and residence, which shall be distinctly announced from the chair.

ARTICLE IV.—MEETINGS.

The regular meetings of the Association shall be held annually, and commence on the last Tuesday in July. The place of meeting shall be determined each year by vote of the association.

ARTICLE V.—OFFICERS.

SEC. 1.—The officers of the Association shall be a President, two Vice-Presidents, Corresponding Secretary, Recording Secretary, and a Treasurer. Two or more nominees for each office shall be presented by a special committee of one from each delegation, and shall be voted for separately by ballot, a plurality of votes being necessary for an election. Each officer shall hold his appointment for one year, and until another is elected to succeed him.

SEC. 2.—The President shall preside at the meetings, and perform all the duties that custom or parliamentary usage may require.

SEC. 3.—*The Vice-President.* In the absence of the President, one of the Vice-Presidents shall assume all the duties of the office; and in the absence of these officers, a chairman *pro tem.* shall be appointed *viva voce.*

SEC. 4.—The *Corresponding Secretary* shall attend to the correspondence with the Association, and give due notice of the time and place of each annual meeting.

SEC. 5.—The *Recording Secretary* shall keep accurate minutes of the proceedings of the Association, preserve the archives and unpublished transactions, and attend to all other duties that appertain to the office.

ARTICLE VI.—STANDING COMMITTEES.

SEC. 1.—The following Standing Committees shall be organized at every annual meeting, to prepare, arrange and expedite business for each ensuing year, and to carry into effect the orders of the Association, not otherwise assigned, namely: a Committee of Arrangements, of Publication, on Prize Essays, on Dental Physiology, on Dental Chemistry, on Dental Pathology and Surgery, on Mechanical Dentistry, Dental Education, and Dental Literature.

SEC. 1.—The *Committee of Arrangements* shall be composed of three members, one of whom, if practicable, shall reside in the place at which the Association is to hold its next ensuing annual meeting. They shall be required to procure suitable accommodations for the meeting; to verify and report upon the credentials of membership; to receive and announce all essays and memoirs voluntarily communicated, either by members of the Association, or by others through them; and to select and announce, at the earliest period practicable after their appointment, subjects for discussion at the succeeding annual meeting.

SEC. 2.—The *Committee of Publication* shall consist of five members, of which the *Corresponding Secretary*, and *Treasurer* must constitute a part. They shall be authorized to employ a competent reporter to furnish an accurate report of the proceedings of each meeting. They shall superintend the publication and distribution of such portions of the Transactions as the Association may direct. This Committee shall audit the accounts of the Treasurer, and present a statement of the same in the annual report of the Committee, which report shall specify the character and cost of the publications of the Association during the year; the number of copies still at the disposal of the me^ting, and the funds on hand for further operations. The Committee shall be instructed to print, at the beginning of each volume of the Transactions, the followiug disclaimer, viz: "The American Dental Association, although formally accepting and publishing the report of the various standing committees, and essays read before the Association, holds itself wholly irresponsible for the opinions, theories, or criticisms therein contained, except when so decided by special resolution."

SEC. 3.—The *Committee on Prize Essays* shall consist of five members. Two prizes, consisting of medals not exceeding in value fifty dollars each, may be awarded to the best two communications reported on favorably by the Committee, and directed by the Association to be published.

SEC. 4.—The *Committee on Dental Physiology, and Dental Chemistry* shall be composed of three members each. It shall be the duty of each Committee to present yearly reports in their special departments, embracing, if practicable, the results of original investigations.

SEC. 5.—The *Committee on Dental Pathology and Surgery*, consisting of five members, shall have under consideration every thing that appertains to pathological conditions of the teeth and adjacent tissues, and the remedial agencies embraced under the head of operative dentistry. Improvements in the latter department, if expedient, shall be thoroughly tested, and reported on before the Association by this Committee.

SEC. 6.—The *Committee on Mechanical Dentistry* shall consist of five members, who shall receive and take cognizance of plans, improvements and specimens in this department, that may be presented by persons desiring to bring them before the Association. The Committee shall be authorized to reject those they may deem unworthy of presentation.

Sec. 7.—The *Committee on Dental Education, and Dental Literature*, consisting each of three members, shall make annual reports on these important subjects. The selection of the chairman and members of the different committees, shall be referred to the nominating committee.

ARTICLE VII.—AMENDMENTS.

These articles may be altered and amended with the consent of three-fourths of the members present, at the subsequent annual meeting to that at which such amendment or alteration may have been proposed.

### THE ORDER OF BUSINESS.

The order of business at the annual meeting of the American Dental Association, shall at all times be subject to the vote of three-fourths of all the members in attendance; and until permanently altered, except when for a time suspended, it shall be as follows, viz:

1. The temporary organization of the meeting, preparatory to the election of officers.
2. The report of the Committee of Arrangements, on the credentials of members.
3. The calling of the roll.
4. The reading of minutes.
5. The election of officers.
6. The reception of members not present at the opening of the meeting, and the reading of notes from absentees.
7. The reading and consideration of the stated annual reports from the standing committees.
8. The reading of essays.
9. The discussion of topics selected for the session.
10. The selection of the place of next annual meeting.
11. The appointment of standing committees.
12. Resolutions, introducing new business, and instructions to the permanent committees.
13. Unfinished and miscellaneous business.
14. Adjournments.

During the discussions upon the Constitution, a paper from the St. Louis Dental Association, containing some suggestions in regard to the Constitution, was offered by Dr. Blake. That Association having no delegate present, it was concluded to hear the suggestions; the paper was read by Dr. Blake.

Adjourned to meet to-morrow, at 10 o'clock, A. M.

WEDNESDAY, AUG. 1.—10 O'CLOCK, A. M.

The Association met according to adjournment.

The record of the proceedings of yesterday was read and adopted.

On motion of Dr. H. R. Smith,

*Resolved*, That this Association extend an invitation to members of the profession and others of Washington City, who may desire to be present during the deliberations.

On motion,

*Resolved*, That the Association now proceed to the election of officers for the ensuing year.

On motion of Dr. M'Quillen,

*Resolved*, That we now take a recess of fifteen minutes, that the various delegations may each select their member of the Nominating Committee.

On call, the following persons were found to constitute that committee, viz:

Drs. J. B. Gibbs, Washington City Dental Association; F. Y. Clark, Georgia Dental Association, Savannah; H. R. Smith, Cincinnati Dental Society; Wm. A. Pease, Mad River Valley Dental Association; J. F. Flagg, Penn. Association of Dental Surgeons; J. L. Suesserott, Penn. College of Dental Surgery; A. Blake, Western Dental Society; P. G. C. Hunt, Indiana State Dental Association; W. Muir Rogers, Kentucky State Dental Association; W. H. Atkinson, Mississippi Valley Dental Association.

The committee retired, and after due consultation and deliberation, made the following report:

Your committee would report that the following nominations have been made, viz :

Dr. W. H. Atkinson, *President.*

" J. B. Gibbs, 1*st Vice President.*

" W. Cahoon, 2*d* "

" J. Taft, *Recording Secretary.*

" W. Muir Rogers, *Corresponding Secretary.*

" S. Dillingham, *Treasurer.*

On motion,

*Resolved*, That a vote now be taken on the nominees presented by the committee.

Which being done, the nominees were found to be duly elected.

On motion,

*Resolved*, That the nomination and election of the various standing committees be postponed till the afternoon session.

The Presioent appointed Drs. Rogers and McQuillen to conduct the President elect to the chair.

On motion of Dr. Fitch,

*Resolved*, That we reconsider the resolution adopting the Constitution.

*Resolved*, That the fifth section of the Constitution be amended to read as follows : The officers of this Association shall be a President, two Vice Presidents, Corresponding Secretary, Recording Secretary, and Treasurer. Two or more nominees for each office shall be presented by the nominating committee ; said committee shall consist of one member from each delegation, and shall be voted for separately by ballot ; a plurality of votes being necessary for an election.

Adjourned to meet at 4 o'clock, P. M.

4 o'clock, p. m.

The Association met according to adjournment. The President in the chair.

The Minutes of the morning session were read and adopted.

On motion,

*Resolved*, That the thanks of the Association be tendered to Dr. W. W. Allport, as a testimonial of its appreciation of the able manner in which he has discharged his duties as President of this Association.

*Resolved*, That the Secretary be instructed to procure two books, one for the Constitution, By-laws, and signatures of the members; and one for the records of the Association.

On motion,

*Resolved*, That the Secretary be empowered to employ a competent person to transcribe the Constitution, as adopted, into a book suitable for the purpose, that the members may have an opportunity to sign the same.

On motion of Dr. Cahoon,

*Resolved*, That a committee of three be appointed to draft a Code of By-Laws for this Association.

The President appointed for this committee Drs. Cahoon, Allport and Suesserott.

The committee were requested to report at the earliest practicable period.

A paper from Dr. A. M. Moore, of Indiana, was received, read and ordered to be placed on file; and the thanks of the Association were tendered to Dr. Moore for his suggestions.

A paper was read by Dr. A. Blake, giving a history of the formation of the Western Dental Association, which was ordered to be placed on file.

On motion of Dr. W. M. Rogers,

*Resolved*, That this Association recommend to all dental practitioners throughout the Union the propriety of urging all students under their tuition to prosecute a Collegiate Dental education. Also, that we feel the importance and necessity of a high standard of literary and scientific attainments by the dental students.

A very spirited discussion arose upon this resolution, which elicited the free expression of opinion from all the members present.

Adjourned to 9 o'clock, A. M., to-morrow.

THIRD DAY.—9 O'CLOCK, A. M.

The Association met according to adjournment; the Minutes of the last session were read and approved.

The reading of essays being now in order,

Dr. J. F. FLAGG read a paper on the claims of dentistry on dentists. It was received and ordered to be placed in the hands of the publishing committee.

Dr. FITCH presented and read a paper on extraction of the teeth, which was received and ordered to be placed in the hands of the publishing committee.

Dr. GEO. F. BARKER presented and read a paper upon the demands of the dental profession. It was also received and placed in the hands of the publishing committee.

Dr. TAFT read a paper upon the buccal secretions. It was also ordered to be placed in the hands of the publishing committee.

Dr. M'QUILLEN presented and delivered a synopsis of a of a paper upon the anatomy, physiology, pathology and remedial treatment of the fifth pair of nerves. Illustrations were given from a fine model.

The Committee on By-Laws was called upon, and made the following report:

Your committee would most respectfully submit the following By-Laws for your consideration, viz:

### BY-LAWS.

### I. OF THE PRESIDENT.

The President shall preside at all meetings, keep order, state and put questions, regulate debates, and sign all orders on the Treasurer, duly passed by the society. He shall not discuss any question while in the chair, unless it be a question of order. He shall have no vote, except when his vote may be necessary to decide a question upon a call of yeas and nays, or upon a ballot, except in the ballot for officers.

### II. OF MEMBERS.

Each delegate, prior to being considered a member of this Association, shall sign the Constitution and pay the annual assessment.

III. CONDUCT OF MEMBERS.

Any act of special immorality or unprofessional conduct, committed by a member of this Association, shall be referred to the Committee of Arrangements, whose duty it shall be to thoroughly examine into the case and report at the next meeting, if the charges be sustained. Whereupon, by vote, the offending member may be reprimanded or expelled, a two-thirds vote being required for expulsion, a plurality being sufficient for reprimand.

IV. DEBATE.

No member shall be permitted to address the chair more than twice upon the same subject,—nor shall he consume more than fifteen minutes, unless by consent of the Association.

ADDITIONS TO BY-LAWS.

These ByLaws may be amended or added to by a vote of two-thirds of the members present at any regular meeting.

In acknowledgement of having adopted the foregoing propositions, and of our willingness to abide by them, and use our endeavors to carry into effect the objects of this Association, as above set forth, we have hereunto set our names:

S. Dillingham, J. H. McQuillen, C. P. Fitch, Geo. T. Barker, J. Foster Flagg, J. W. Van Osten, Philadelphia, Penn. Dental Association.

Danl. McFarlan, Washington City, Pa. Dental Association.

B. M. Gildea, Harrisburg, Penn. Dental Association.

J. L. Suesserott, Penn. Dental Association.

Wm. Cahoon, Detroit, Mich. Dental Association.

A. S. Talbert, Lexington, Ky., Kentucky State Dental Association.

W. M. Rogers, Shelbyville, Ky., Kentucky State Dental Association.

F. Y. Clarke, Savannah, Ga., Georgia State Dental Society.

W. A. Pease, Dayton, O., Mad River Valley Dental Association.

A. Blake, St. Louis, Mo., Western Dental Society.

J. Taft, H. R. Smith, Cincinnati, O., Cincinnati Dental Society.

W. H. Atkinson, Cleveland, O., Miss. Valley Association.

J. B. Gibbs, H. N. Wadsworth, Washington, D. C., Washington Dental Association.

W. W. Allport, Chicago, Ill., Western Dental Society.

P. G. C. Hunt, S. B. Smith, Terre Haute, Ind., Indiana State Dental Association.

On motion of Dr. Allport,

*Resolved*, That the American Dental Association most respectfully recommend to our Dental Colleges the necessity of being more particular in requiring of their matriculants a higher order of general literary and scientific education; and the strictest enforcement of their standard requirements for graduation.

On motion of Dr. Van Osten,

*Resolved*, That a committee of three be appointed to confer with dental practitioners throughout the Union, with a view to the formation of local Societies, and to report to this Association, at its next stated meeting.

The Chair appointed upon that committee, Drs. Van Osten, Allport and Rogers.

Adjourned to 4 o'clock, P. M.

4 O'CLOCK, P. M.

Association met according to adjournment. The members signed the Constitution and paid dues.

The Committee on Nominations made the following report:

*Committee of Arrangements*—Drs. Cahoon, Atkinson and Fitch.

*Committee on Publication*—Drs. Taft, Rogers, Allport, Dillingham and Van Osten.

*Committee on Prize Essays*—Drs. Hunt, Gildea, Clark, Rogers and Suesserott.

*Committee on Dental Physiology*—Drs. M'Quillen, Barker and M'Farlan.

*Committee on Dental Pathology and Surgery*—Drs. Suesserott, Atkinson, Flagg, Hunt and Allport.

*Committee on Mechanical Dentistry*—Drs. S. B. Smith, Blake, Talbert, Dillingham and Wordsworth.

*Committee on Dental Education*—Drs. Rogers, Fitch and Hunt.

*Committee on Dental Literature*—Drs. Barker, Gibbs and Taft.

*Committee on Dental Chemistry*—Drs. Pease, Taft and Van Osten.

These nominations were confirmed by the Association.

On motion,

*Resolved* That the next annnal meeting of the American Dental Association be held in the City of Cleveland, Ohio.

Prof. BRAINARD, of Cleveland, Ohio, was introduced to the Association, and made some very interesting remarks upon Chemistry as connected with the teeth.

On motion,

*Resolved*, That the thanks of the Association be tendered to the Regents of the Smithsonian Institute; and that the Recording Secretary be instructed to pay the Janitor's fees, and any other indebtedness that may have accrued in taking care of the Hall during the sessions of this Association.

Adjourned to meet in the City of Cleveland, Ohio, on the last Tuesday of July, 1861, at 10 o'clock, A. M.

J. TAFT, *Sec'y.*

---

CINCINNATI, Tuesday Evening, Sept. 11, 1860.

Local Dental Association met pursuant to adjournment, at the office of Drs. Bonsall & Smith. Members present—Drs. Taft, Foote, Bonsall, Wells, Davenport, Wheeler, and H. A. Smith.

Minutes of last meeting read and approved.

Dr. Foote read an Essay—Subject: Dental Associations.

Dr. Richardson's appointment as Essayist was continued.

This being the regular evening for election of officers, the following gentlemen were elected to the respective offices, viz:

*President*—H. A. Smith.

*Vice President*—B. D. Wheeler.

*Recording Secretary*—T. F. Davenport.

*Corresponding Secretary*—J. Taft.

*Treasurer*—Chas. Bonsall.

*Publishing Committee*—Drs. Richardson, Davenport and Wheeler.

Dr. Taft, the retiring President, read a paper, after which, Dr. Smith, President elect, took the chair.

Dr. Wheeler was appointed Essayist for the next meeting, in connection with Dr. Richardson, continued.

Subject for discussion at next meeting—*Tooth-ache.*

Adjourned to meet at Dr. Davenport's office on the second Tuesday in October. T. F. DAVENPORT, *Rec. Sec'y.*

